# Autocrine IGF-I/insulin receptor axis compensates for inhibition of AKT in ER-positive breast cancer cells with resistance to estrogen deprivation

**DOI:** 10.1186/bcr3449

**Published:** 2013-07-11

**Authors:** Emily M Fox, María Gabriela Kuba, Todd W Miller, Barry R Davies, Carlos L Arteaga

**Affiliations:** 1 Department of Medicine, Vanderbilt-Ingram Cancer Center, Vanderbilt University, 2220 Pierce Ave, 777 PRB, Nashville, TN 37232-6307, USA; 2 Department of Pathology, Microbiology & Immunology, Vanderbilt-Ingram Cancer Center, Vanderbilt University, 2220 Pierce Ave, 777 PRB, Nashville, TN 37232-6307, USA; 3 Department of Pharmacology and Toxicology, Norris Cotton Cancer Center, Geisel School of Medicine at Dartmouth, One Medical Center Drive, HB-7936, Lebanon, NH 03756, USA; 4 Oncology Innovative Medicine, AstraZeneca, Alderley Park, Macclesfield, SK0 4TG, UK; 5 Department of Cancer Biology, Vanderbilt-Ingram Cancer Center, Vanderbilt University, 2220 Pierce Ave, 777 PRB, Nashville, TN 37232-6307, USA; 6 Breast Cancer Research Program, Vanderbilt-Ingram Cancer Center, Vanderbilt University, 2220 Pierce Ave, 777 PRB, Nashville, TN 37232-6307, USA

**Keywords:** AKT, ER+ breast cancer, endocrine resistance, IGF-IR, InsR

## Abstract

**Introduction:**

Estrogen receptor α-positive (ER+) breast cancers adapt to hormone deprivation and acquire resistance to antiestrogen therapies. Upon acquisition of hormone independence, ER+ breast cancer cells increase their dependence on the phosphatidylinositol-3 kinase (PI3K)/AKT pathway. We examined the effects of AKT inhibition and its compensatory upregulation of insulin-like growth factor (IGF)-I/InsR signaling in ER+ breast cancer cells with acquired resistance to estrogen deprivation.

**Methods:**

Inhibition of AKT using the catalytic inhibitor AZD5363 was examined in four ER+ breast cancer cell lines resistant to long-term estrogen deprivation (LTED) by western blotting and proliferation assays. Feedback upregulation and activation of receptor tyrosine kinases (RTKs) was examined by western blotting, real-time qPCR, ELISAs, membrane localization of AKT PH-GFP by immunofluorescence and phospho-RTK arrays. For studies *in vivo*, athymic mice with MCF-7 xenografts were treated with AZD5363 and fulvestrant with either the ATP-competitive IGF-IR/InsR inhibitor AZD9362 or the fibroblast growth factor receptor (FGFR) inhibitor AZD4547.

**Results:**

Treatment with AZD5363 reduced phosphorylation of the AKT/mTOR substrates PRAS40, GSK3α/β and S6K while inducing hyperphosphorylation of AKT at T308 and S473. Inhibition of AKT with AZD5363 suppressed growth of three of four ER+ LTED lines and prevented emergence of hormone-independent MCF-7, ZR75-1 and MDA-361 cells. AZD5363 suppressed growth of MCF-7 xenografts in ovariectomized mice and a patient-derived luminal B xenograft unresponsive to tamoxifen or fulvestrant. Combined treatment with AZD5363 and fulvestrant suppressed MCF-7 xenograft growth better than either drug alone. Inhibition of AKT with AZD5363 resulted in upregulation and activation of RTKs, including IGF-IR and InsR, upregulation of FoxO3a and ERα mRNAs as well as FoxO- and ER-dependent transcription of IGF-I and IGF-II ligands. Inhibition of IGF-IR/InsR or PI3K abrogated AKT PH-GFP membrane localization and T308 P-AKT following treatment with AZD5363. Treatment with IGFBP-3 blocked AZD5363-induced P-IGF-IR/InsR and T308 P-AKT, suggesting that receptor phosphorylation was dependent on increased autocrine ligands. Finally, treatment with the dual IGF-IR/InsR inhibitor AZD9362 enhanced the anti-tumor effect of AZD5363 in MCF-7/LTED cells and MCF-7 xenografts in ovariectomized mice devoid of estrogen supplementation.

**Conclusions:**

These data suggest combinations of AKT and IGF-IR/InsR inhibitors would be an effective treatment strategy against hormone-independent ER+ breast cancer.

## Introduction

AKT is a serine/threonine kinase downstream of phosphatidylinositol-3 kinase (PI3K) that plays a critical role in cellular survival, proliferation, metabolism and resistance to apoptosis [1]. Upon activation by growth factor receptor tyrosine kinases (RTKs) and G-protein-coupled receptors, PI3K phosphorylates phosphatidylinositol 4,5-bisphosphate (PIP_2_) to produce phosphatidylinositol 3,4,5-trisphosphate (PIP_3_). PIP_3 _then recruits pleckstrin homology (PH) domain-containing proteins such as PDK1, SGK and AKT to the plasma membrane, where AKT is phosphorylated at T308 by PDK-1 and, subsequently, at S473 by TORC2, becoming fully activated [1,2].

The PI3K/AKT signaling pathway is the most frequently mutated pathway in breast cancer [2-4]. PI3K is activated via several mechanisms, including gain-of-function mutations in the PI3K catalytic subunit p110α (*PIK3CA*) and regulatory subunit p85α (*PIK3R1*), amplification of wild type PIK3CA, p110β (*PIK3CB*) and PDK1, loss/inactivation of the PIP3 phosphatases PTEN and INPP4B, mutation and/or amplification of AKT1-3 and amplification of RTKs, such as HER2, IGF-IR, MET, FGFR1 and EGFR [3,5]. These cumulative data have suggested AKT as a rational molecular target for breast cancer therapy.

About 80% of breast cancers express estrogen receptor α (ER) and/or progesterone receptor (PR), biomarkers indicative of hormone dependence [6]. Therapies against ER+ breast cancers inhibit ER function either by antagonizing ligand binding to ER (tamoxifen), downregulating ER (fulvestrant) or blocking estrogen biosynthesis (aromatase inhibitors (AIs)). However, many tumors exhibit *de novo *or acquired resistance to endocrine therapies. Overexpression of the *ErbB2/HER2 *protooncogene has been shown to promote clinical resistance to antiestrogen therapy [7,8]. However, <10% of ER+ breast cancers overexpress HER2, suggesting that, for the majority of ER+ breast cancers, mechanisms of escape from endocrine therapy remain to be discovered.

The PI3K pathway has been causally associated with resistance to endocrine therapy [9-14]. Upon acquisition of hormone independence, ER+ breast cancer cells increase their dependence on PI3K/AKT signaling [9]. Herein we show that inhibition of AKT using the catalytic inhibitor AZD5363, currently in phase I clinical trials, suppressed hormone-independent ER+ breast cancer growth. However, upregulation of IGF-IR/InsR and their ligands compensated for AKT inhibition and limited the effect of AZD5363. Addition of an IGF-IR/InsR tyrosine kinase inhibitor (TKI) enhanced the action of AZD5363 against MCF-7 xenografts in ovariectomized mice devoid of estrogen supplementation, suggesting a novel and testable therapeutic combination for patients with ER+ breast cancer.

## Methods

### Cell lines

Cell lines (ATCC, Manassas, VA, USA) were maintained in improved minimum essential medium (IMEM)/10% fetal bovine serum (FBS) (Life Technologies, Grand Island, NY, USA) and authenticated by short tandem repeat profiling using Sanger sequencing (sequenced in March 2011). Long-term estrogen deprived (LTED) cells were generated and maintained in phenol red-free IMEM with 10% dextran/charcoal-treated FBS (DCC-FBS) [9].

### Immunoblot analysis and RTK arrays

Lysates from cells treated with AZD5363 [15], IGF-I, IGF-II, IGFBP-3 (R&D Systems, Minneapolis, MN, USA), AEW541 [16] or BKM120 [17] (Selleck Chemicals, Houston, TX, USA) were subjected to SDS-PAGE, transferred to nitrocellulose and analyzed by immunoblot analysis [9] using antibodies against P-AKT_S473_, P-AKT_T308_, AKT, P-PRAS40, P-GSK-3α/β, P-S6_S240/244_, S6, P-IGF-IRβ_Y1131_/P-InsRβ_Y1146_, P-HER3_Y1197_, P-HER2_Y1248_, P-Src_Y416_, P-FRS2-α_Y436_, EGFR (Cell Signaling, Danvers, MA, USA), InsRβ, IGF-IRβ, ERα (F-10), HER3, HER4, FGFR2 (Santa Cruz Biotechnology, Dallas, TX, USA), HER2 (NeoMarkers, Fremont, CA, USA), PR (Dako, Carpinteria, CA, USA), IRS-1 (EMD Millipore, Billerica, MA, USA), and actin (Sigma-Aldrich, St. Louis, MO, USA). Densitometric analysis was performed using ImageJ. Phospho-RTK arrays were performed using the Human Phospho-RTK Array Kit according to the manufacturer's protocol (R&D Systems).

### Cell proliferation

Cells seeded in triplicate in 12-well plates (2.5 × 10^4 ^cells/well for MCF-7/LTED and 4 × 10^4 ^cells/well for other lines) were treated in 10% DCC-FBS ± AZD5363, selumetinib (AZD6244, ARRY-142886) [18] (Selleck Chemicals), fulvestrant (ICI 182780, R&D Systems), 17β-estradiol (E2) or AZD9362 (AstraZeneca, Cambridge, MA, USA). AZD9362 is a reversible, ATP-competitive small molecule inhibitor of IGF-IR and insulin receptor. In isolated enzyme assays, it inhibits the IGF-IR enzyme with an IC_50 _of 14 nM. In cellular assays, the compound prevents autophosphorylation of IGF-IR in fibroblasts from IGF-IR knockout mice stably transfected with human IGF-IR with an IC_50 _of 48 nM; it inhibits autophosphorylation of human InsR in CHO-T cells with an IC_50 _of 186 nM. AZD9362, dosed at 25 mg/kg qd, also inhibits phosphorylation of IGF-IR by >50% for at least six hours and induces >70% inhibition of tumor volume in NIH3T3 fibroblasts stably transfected with IGF-IR. Media and inhibitors for proliferation assays were replenished every three days; after five to ten days, adherent cells were trypsinized and counted using a Coulter Counter or fixed/stained with crystal violet [9]. For siRNA experiments, cells were transfected in 100-mm dishes using HiPerfect Transfection Reagent according to the manufacturer's protocol (Qiagen, Germantown, MD, USA). The next day, cells were re-seeded in 10% DCC-FBS for immunoblot analyses as described previously [9] or cell proliferation assays and counted five to ten days later. siRNAs targeting *IGF-IR, InsR, HER3*, or non-silencing control were obtained from Qiagen.

### Real-time qPCR

Cells grown in 10% DCC-FBS ± AZD5363 were harvested and their RNA extracted using the RNeasy Mini Kit (Qiagen). Using the iScript cDNA Synthesis Kit (Bio-Rad, Hercules, CA, USA), 1 µg of RNA was reverse transcribed to cDNA and real-time PCR reactions were conducted in 96-well plates using the iCycler iQ (Bio-Rad) and primers obtained from SABiosciences (Qiagen). For siRNA experiments, cells were transfected with siRNA targeting forkhead box class O (*FoxO3) *(Life Technologies)*, ER *or non-silencing control (Qiagen) using Dharmafect 1 according to the manufacturer's protocol (Thermo Fisher Scientific, Pittsburgh, PA, USA). Two days later cells were treated with 10% DCC-FBS ± 2 µM AZD5363 for 24 hours followed by RNA isolation and RT qPCR.

### Confocal microscopy

MCF-7/LTED cells plated in 35-mm dishes with No. 1.5 coverglass coated with Poly-d-lysine (MatTek, Ashland, MA, USA) were transfected with 2.5 µg of an AKT-PH-GFP plasmid (provided by Dr. Gordon Mills, MD Anderson Cancer Center, Houston, TX, USA) using Lipofectamine 2000 according to the manufacturer's protocol (Invitrogen, Life Technologies, Grand Island, NY, USA). On day four, cells were treated with 10% DCC-FBS ± AZD5363, AEW541 or BKM120 for four hours. Cells were viewed on an LSM 510Meta confocal microscope at 40x magnification at the Vanderbilt University Cell Imaging Shared Resource.

### Mouse xenograft experiments

Animal experiments were approved by the Vanderbilt Institutional Animal Care and Use Committee. Female ovariectomized athymic mice were implanted s.c. with a 14-day-release E2 pellet (0.17 mg; Innovative Research of America, Sarasota, Florida, USA). The next day, 107 MCF-7 cells suspended in IMEM and mixed with matrigel (BD Biosciences, San Jose, California, USA) at 1:1 ratio were injected s.c. into the right flank of each mouse. After >2 weeks, mice bearing tumors ≥150 mm^3 ^were randomized to treatment with vehicle (25% (2-hydroxypropyl)-β-cyclodextrin), AZD5363 (150 or 100 mg/kg bid p.o.), fulvestrant (5 mg/week i.p.), AZD9362 (25 mg/kg/day p.o.) or AZD4547 [19] (12.5 mg/kg/day p.o.). Combining 150 mg/kg/day AZD5363 with AZD9362 and AZD4547 resulted in excessive toxicity, so a lower dose of AZD5363 (100 mg/kg/day p.o.) was used in this experiment. Tumor diameters were measured twice weekly and volume in mm^3 ^calculated as volume = width^2 ^x length/2. Tumors were harvested one or four hours after the last dose of AZD5363 or 24 hours after the last dose of fulvestrant and flash-frozen in liquid nitrogen or fixed in 10% formalin prior to paraffin-embedding. Frozen tumors were homogenized using the TissueLyser II (Qiagen). Tumor lysates were prepared, subjected to SDS-PAGE, transferred to nitrocellulose and analyzed by immunoblot analysis [9].

### Statistics

In cell proliferation assays, significant differences were determined by one-way analysis of variance (ANOVA) (one cell line) or two-way ANOVA with Bonferroni post-hoc tests corrected for multiple comparisons. Unpaired *t-*tests were used to determine significant differences in crystal violet assays and real-time qPCR assays. Two-way ANOVA with Bonferroni post-hoc tests corrected for multiple comparisons was used to determine significance in real-time qPCR assays comparing multiple cell lines. In tumor growth assays, significant differences were determined by unpaired *t-*tests. Significant differences in immunohistochemistry (IHC) histoscores were determined by unpaired *t-*tests. *P *<0.05 was considered significant.

[See Additional file 1 for Supplementary materials and methods.]

## Results

### Inhibition of AKT suppresses hormone-independent breast cancer cell growth

**Figure 1:**
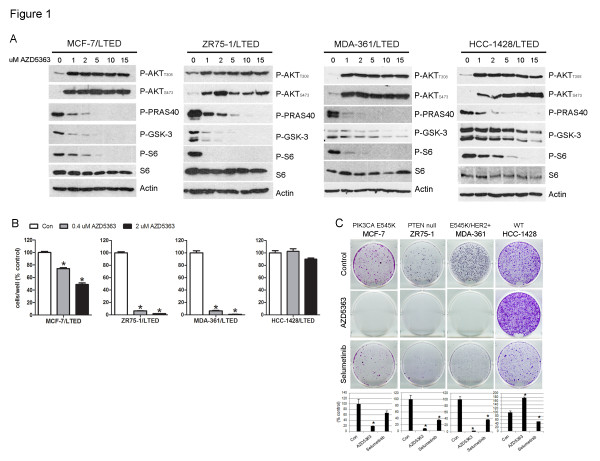
**Catalytic AKT inhibitor suppresses LTED growth and prevents emergence of hormone-independent breast cancer cells**. **A**) LTED cells were treated with 10% DCC-FBS ± 0 to 15 µM AZD5363 for 24 hours. Protein lysates were analyzed by immunoblot using the indicated antibodies. **B**) LTED cells were treated with 10% DCC-FBS ± 0.4 or 2 µM AZD5363. Media and drugs were replenished every three days. Cells were counted after five to ten days. Data are presented as percent of control; each bar, mean ± SEM (*n *= 3; *P <0.0001 versus Con, one-way ANOVA). **C**) Parental cells in 10% DCC-FBS were treated with ± 2 µM AZD5363 or 1 µM selumetinib. Media and drugs were replenished every three days. When control monolayers reached 60% to 80% confluency (after 15 (MCF-7), 36 (ZR75-1 and MDA-361) or 38 (HCC-1428) days, respectively), cells were fixed and stained with crystal violet. Representative images and quantification of integrated intensity (% control) are shown (*P <0.05 versus control, *t*-test). ANOVA, analysis of variance; DCC-FBS, dextran/charcoal-treated fetal bovine serum; LTED, long-term estrogen deprivation; SEM, standard error of the mean.

We previously established a panel of ER+ breast cancer cell lines with acquired resistance to LTED [9]. Treatment with the ATP-competitive AKT inhibitor AZD5363 [15] reduced phosphorylation of the AKT/TORC1 substrates PRAS40, GSK-3α/β and S6K while inducing hyperphosphorylation of AKT in S473 and T308 (Figure 1A). Similar results were seen in MCF-7, ZR75-1 and HCC-1428 parental cells [see Additional file 2, Figure S1]. Catalytic inhibitors of AKT block the activity of the enzyme but release compensatory feedback leading to activation of PI3K and more formation of PIP3 at the membrane. Thus, these compounds do not prevent the recruitment of AKT, via its PH domain, to PIP3 at the plasma membrane. Upon reactivation of PI3K and PIP3 formation, AKT is recruited to the plasma membrane where PDK1 and TORC2 phosphorylate T308 and S473, respectively [20]. As a result, in cells treated with AZD5363, AKT is (hyper)phosphorylated but catalytically inactive (Figure 1A, Figure S1 in Additional file 2; ref [15]). Inhibition of AKT with ≤2 µM AZD5363 suppressed the growth of three of the four LTED lines (Figure 1B). To determine whether AKT is required for the emergence of hormone-independence, we reselected parental cells in estrogen-free medium. Treatment with AZD5363 (0.4 to 2 µM) prevented or delayed the emergence of hormone-independent MCF-7, ZR75-1 and MDA-361 cells (Figure 1C; Additional file 2, Figure S2). Notably, all three of these cell lines contain PI3K pathway alterations (MCF-7 and MDA-361, PIK3CA mutation; ZR75-1, PTEN null), whereas the unresponsive HCC-1428 line does not. In comparison, inhibition of MEK1/2 with selumetinib (AZD6244) [18] induced a more modest inhibition of colony formation in three of the four cell lines tested (Figure 1C; Figure S2 in Additional file 2). AZD5363 also suppressed E2-induced growth in monolayer (data not shown).

### Combined inhibition of AKT and ER suppresses growth of MCF-7 xenografts

**Figure 2:**
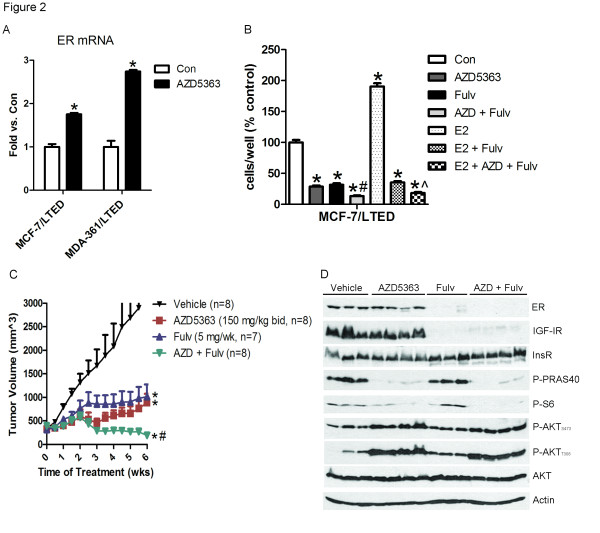
**Combined inhibition of AKT and ER suppresses hormone-independent tumor growth**. **A**) LTED cells were treated with 10% DCC-FBS ± 2 µM AZD5363 for 24 hours. RNA was extracted, reverse transcribed to cDNA and analyzed by real-time PCR. Threshold cycle values were normalized for actin. Data are presented as fold versus control; each bar, mean ± SEM (*n *= 2; *P <0.0001 versus Con, two-way ANOVA). B) LTED cells were treated with 10% DCC-FBS ± 2 µM AZD5363, 1 µM fulvestrant or 1 nM E2. Media and drugs were replenished every three days. Cells were counted after five days. Data are presented as percent of control; each bar, mean ± SEM (*n *= 3; *P <0.0001 versus Con or E2; #*P *<0.01 versus AZD or fulvestrant; ^*P *<0.01 versus E2 + fulvestrant, one-way ANOVA). **C**) MCF-7 cells were injected s.c. into athymic mice supplemented with 14-day release 17β-estradiol pellets. Mice bearing tumors ≥150 mm^3 ^were randomized to vehicle, AZD5363 (150 mg/kg/day bid), fulvestrant (5 mg/wk), or AZD5363 + fulvestrant for six weeks. Data are presented as mean tumor volume ± SEM (*P <0.01 versus vehicle; ^#^*P *<0.001 versus AZD or fulvestrant, *t*-test). **D**) Xenografts from C) were homogenized one hour after the last dose and tumor lysates were analyzed by immunoblot using the indicated antibodies. ANOVA, analysis of variance; DCC-FBS, dextran/charcoal-treated fetal bovine serum; LTED, long-term estrogen deprivation; SEM, standard error of the mean.

Upon escape from hormone deprivation, some ER+ tumor cells retain estrogen (ligand)-independent ER function. PI3K/AKT can phosphorylate and activate ER transcription in the absence of estradiol [12]. Estrogen deprivation induces synthetic lethality in ER+ breast cancer cells treated with a PI3K inhibitor or transfected with p110 siRNA [13], suggesting compensatory crosstalk between ER and PI3K/AKT signaling. Consistent with this crosstalk, inhibition of AKT with AZD5363 resulted in upregulation of ER mRNA in LTED lines (Figure 2A). We also saw upregulation of ER protein and its transcriptional target PR in T47D, MCF-7 and MDA-361 cells following treatment with the pan-PI3K inhibitor BKM120 [17] [see Additional file 2, Figure S3), suggesting that this feedback also occurs with other inhibitors of the PI3K/AKT pathway. Treatment with the ER downregulator fulvestrant significantly enhanced the growth-inhibitory effects of AZD5363 in MCF-7/LTED cells, and addition of AZD5363 significantly enhanced the ability of fulvestrant to block estrogen-induced cell proliferation (Figure 2B).

We next assessed the effects of the AKT inhibitor ± fulvestrant on tumor growth *in vivo*. ER+/PIK3CA mutant MCF-7 xenografts were established in ovariectomized athymic female mice supplemented with a 14-day-release E2 pellet. The E2 pellets expired after 14 days. Four weeks after tumor cell inoculation, mice with tumors measuring ≥150 mm^3 ^were randomized to treatment with vehicle, AZD5363, fulvestrant or the combination. AZD5363 and fulvestrant significantly inhibited tumor growth compared to vehicle-treated controls (Figure 2C). Treatment with both AZD5363 and fulvestrant suppressed xenograft growth >90%; this effect was statistically better than either drug alone (Figure 2C). AZD5363-treated tumors exhibited lower levels of P-PRAS40 and P-S6 but higher levels of P-AKT compared to control tumors (Figure 2D). Treatment with fulvestrant alone or with both drugs downregulated ER and IGF-IR protein levels. Tumors treated with fulvestrant for six weeks exhibited higher levels of T308 P-AKT (Figure 2D). Finally, addition of AZD5363 enhanced fulvestrant-induced inhibition of tumor cell proliferation as measured by Ki67 IHC [see Additional file 2, Figure S4]. These data suggest that simultaneous inhibition of AKT and ER is more effective than inhibition of each molecular target alone against MCF-7 xenografts *in vivo*. They also imply that AKT and ER inhibitors induce an adaptive response that limits their efficacy as single agents; that is, cells may compensate by signaling with the alternative (spared) pathway when only one pathway is inhibited.

Inhibition of AKT was also effective against other models of endocrine resistance. HBCx-3 ER+ luminal B breast cancer xenografts were established in nude mice after resection from a post-menopausal woman with no previous treatment [21]. These xenografts were negative for PTEN and HER2 protein by IHC [see Additional file 2, Figure S5A]. Although these xenografts were resistant to tamoxifen and fulvestrant, treatment with AZD5363 suppressed tumor growth [see Additional file 2, Figure S5B-C]. Further, AZD5363 treatment increased ER protein levels in the HBCx-3 xenografts [see Additional file 2, Figure S5D], suggesting that active AKT represses ER expression both *in vitro *(Figure 2A; Additional file 2, Figure S3) and *in vivo*.

### Inhibition of AKT results in upregulation of RTKs *in vitro *and *in vivo*

**Figure 3:**
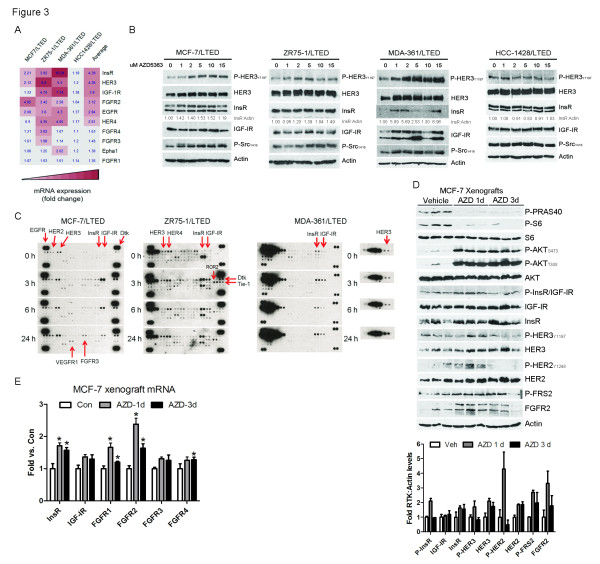
**Inhibition of AKT results in feedback upregulation of RTKs**. **A**) LTED cells were treated with 10% DCC-FBS ± 2 µM AZD5363 for 24 hours. RNA was extracted, reverse transcribed to cDNA and analyzed by real-time PCR. Threshold cycle values were normalized for actin. Average fold changes over control (Con) were calculated and used to generate a heatmap. **B**) LTED cells were treated for 24 hours in 10% DCC-FBS ± 15 µM AZD5363. Protein lysates were analyzed by immunoblot. Densitometric analysis was performed and the ratio of InsR:actin is shown below the InsR blots. **C**) LTED cells in 10% DCC-FBS were treated ± 2 µM AZD5363 for 0 to 24 hours. Cell lysates (0.5 mg) were prepared and analyzed by phospho-RTK arrays. **D**) Mice bearing MCF-7 xenografts ≥150 mm^3 ^were treated with vehicle or AZD5363 (150 mg/kg bid p.o.) for one or three days. Xenografts were harvested four hours after the last dose; tumor lysates were prepared and analyzed by immunoblot. Densitometric analysis was performed and a graphical representation of the average RTK:actin levels is shown below the blot. **E**) Xenografts from D) were homogenized and RNA was extracted and analyzed by real-time PCR as in A. Data are presented as fold versus control; each bar, mean ± SEM (*n *= 3; *P <0.05 versus Con, *t*-test). DCC-FBS, dextran/charcoal-treated fetal bovine serum; LTED, long-term estrogen deprivation; RTK, receptor tyrosine kinase; SEM, standard error of the mean.

We and others have previously reported that inhibition of PI3K/AKT/mTOR induces compensatory expression and activation of several RTKs [22-24]. In order to identify inhibitors that can be rationally combined with the AKT antagonist in hormone-independent breast cancer, we examined the effects of AZD5363 on a set of therapeutically targetable RTKs. Treatment with AZD5363 upregulated mRNA levels of several RTKs, with InsR, HER3 and IGF-IR being the top hits across all four LTED lines (Figure 3A). FGFR 2-4 mRNAs were also induced upon treatment with AZD5363. Inhibition of AKT resulted in upregulation of total and phosphorylated HER3 in three of the four LTED lines, as well as Y416 P-Src protein levels (Figure 3B). Treatment with 2 µM AZD5363 upregulated InsR protein 1.4-fold in MCF-7/LTED cells and 5.7-fold in MDA-361/LTED cells (see densitometric analysis in Figure 3B). Treatment with the Src kinase inhibitor dasatinib [25] decreased AZD5363-induced upregulation of phosphorylated HER3 in MCF-7/LTED cells, as well as significantly enhanced the growth inhibitory effects of AZD5363 [see Additional file 2, Figure S6A-B]. However, treatment with the Src inhibitor AZD0530 [26] was ineffective. Pre-treatment with the IGF-IR/InsR dual TKI AEW541 [27] or BKM120 abrogated the AZD5363-induced increase in P-Src [see Additional file 2, Figure S6C], suggesting the increase in active Src was due to activation of IGF-IR/InsR and PI3K.

We next assessed the effects of AZD5363 on a wider panel of RTKs. Following inhibition of AKT in MCF-7/LTED, ZR75-1/LTED and MDA-361/LTED cells, phospho-RTK array analysis revealed increased phosphorylation of multiple RTKs, including InsR, IGF-IR, HER3, EGFR, HER2, HER4, Dtk, VEGFR1 and FGFR2-4 (Figure 3C; Additional file 2, Figure S7). To validate these findings *in vivo*, we treated ovariectomized mice bearing MCF-7 xenografts with AZD5363 for one or three days. Inhibition of AKT upregulated the tumor levels of P-InsR/IGF-IR, InsR, P-HER3, HER3, P-HER2, HER2, the FGFR substrate P-FRS2 and FGFR2 proteins (Figure 3D). Further, treatment with AZD5363 for one to three days also increased tumor levels of InsR, IGF-IR and FGFR 1-4 mRNAs (Figure 3E).

### Inhibition of IGF-IR/InsR or PI3K abrogates AZD5363-induced AKT membrane localization and phosphorylation

**Figure 4:**
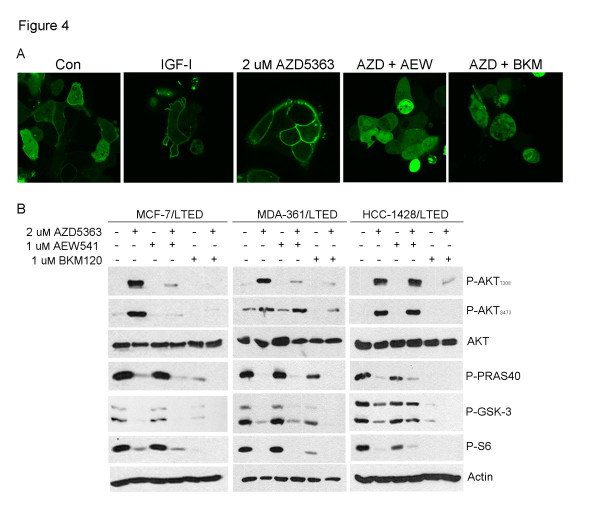
**Inhibition of IGF-IR/InsR or PI3K abrogates AZD5363-induced AKT membrane localization and phosphorylation**. **A**) MCF-7/LTED cells were transfected with an AKT PH-GFP plasmid. On day four, cells were treated with 100 ng/ml IGF-I in serum-free medium for 15 minutes, or pre-incubated with 10% DCC-FBS ± 1 µM AEW541 or 1 µM BKM120 for 30 minutes followed by treatment with 2 µM AZD5363 for four hours. Cells were viewed in a LSM 510Meta confocal microscope at 40x magnification. **B**) LTED lines were treated with 10% DCC-FBS ± 1 µM AEW541 or BKM120 for 1 hour, followed by the addition of 2 µM AZD5363 for 24 hours. Protein lysates were analyzed by immunoblot using the indicated antibodies. DCC-FBS, dextran/charcoal-treated fetal bovine serum; IGF-I, insulin-like growth factor-I; LTED, long-term estrogen deprivation.

We speculated that upregulation of activated InsR/IGF-IR was maintaining PI3K activity and PIP3 formation to counteract the inhibition of AKT and, thus, limit the action of AZD5363. To test this possibility, we transfected MCF-7/LTED cells with a fusion protein comprised of the AKT PH domain fused to the amino-terminus of GFP [24,28]. PIP3 binding to the PH domain should result in translocation of the fusion protein to the plasma membrane. AKT PH-GFP was mainly cytoplasmic in control cells, whereas treatment with exogenous IGF-I induced its translocation to the membrane (Figure 4A). Treatment with AZD5363 also induced marked translocation of AKT PH-GFP to the membrane, suggestive of increased PIP3 production and, as a result, AKT phosphorylation at the T308 PDK-1 site. Pre-treatment with the IGF-IR/InsR TKI AEW541 or BKM120 prevented AZD5363-induced membrane localization of AKT PH-GFP (Figure 4A), as well as abrogated the AZD5363-induced increase in AKT phosphorylation at T308 and S473 in three LTED lines (Figure 4B). Combined treatment with BKM120 and AZD5363 resulted in greater inhibition of P-PRAS40 and P-GSK-3 compared to each inhibitor alone (Figure 4B). Together, these data suggest that following inhibition of AKT in LTED cells, the phosphorylation of AKT is at least in part due to compensatory upregulation of IGF-IR/InsR signaling and PIP3 formation.

### Inhibition of AKT results in FoxO-dependent upregulation of IGF-IR/InsR ligands

**Figure 5:**
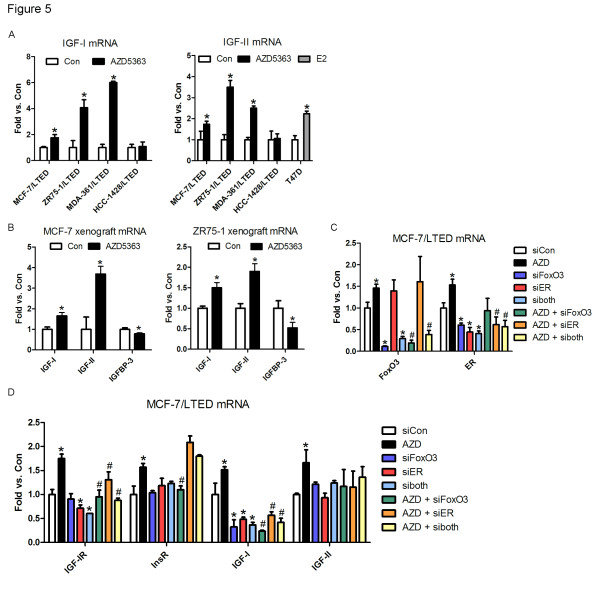
**AZD5363-induced upregulation of IGF-IR, IGF-I and IGF-II is dependent on ER and FoxO3**. **A**) LTED cells were treated with 10% DCC-FBS ± 2 µM AZD5363 for 24 hours. T47D cells were serum starved for 24 hours and then treated with 10% DCC-FBS ± 2 µM AZD5363 for 24 hours. RNA was extracted, reverse transcribed to cDNA and analyzed by real-time PCR. Threshold cycle values were normalized for actin. qPCR reactions with no input RNA were used as negative controls. Data are presented as fold versus control; each bar, mean ± SEM (*n *= 4; *P <0.05 versus Con, *t*-test). **B**) Mice bearing MCF-7 or ZR75-1 xenografts ≥150 mm^3 ^were treated with vehicle or AZD5363 (150 mg/kg bid) for one day. Xenografts were harvested four hours after the last dose and homogenized. RNA was extracted, reverse transcribed to cDNA and analyzed by real-time PCR. Threshold cycle values were normalized for actin (MCF-7) or 36B4 (ZR75-1). Data are presented as fold versus control; each bar, mean ± SEM (*n *= 3; *P <0.05 versus Con, *t*-test). **C, D**) MCF-7/LTED cells were transfected with siRNA targeting FoxO3, ER, both (siboth) or a non-silencing control (siCon). Two days later the cells were treated with 10% DCC-FBS ± 2 µM AZD5363 for 24 hours. RNA was extracted and analyzed by real-time PCR as in A. Data are presented as fold versus control; each bar, mean ± SEM (*n *= 3). C) *P <0.01 versus siCon, *t*-test. D) *P <0.05 versus siCon; #*P *<0.05 versus AZD, *t*-test. DCC-FBS, dextran/charcoal-treated fetal bovine serum; ER, estrogen receptor; FoxO, forkhead box class O; LTED, long-term estrogen deprivation.

We next investigated mechanisms of IGF-IR/InsR phosphorylation upon inhibition of AKT. Treatment with AZD5363 (24 hours) upregulated mRNA levels of IGF-I and IGF-II in three of the four LTED cell lines (Figure 5A), as well as in MCF-7 and ZR75-1 xenografts (Figure 5B). E2 induction of IGF-II mRNA in T47D cells served as a positive control for IGF-II expression (Figure 5A). Treatment with AZD5363 also increased IGF-I and IGF-II protein levels in the cell culture supernatants of three of the four LTED lines [see Additional file 2, Figure S8]. IGF-I and IGF-II bind IGF-IR/InsR heterodimers and IGF-IR homodimers [29]. Of note, short-term treatment of MCF-7 and ZR75-1 xenografts with AZD5363 downregulated mRNA levels of IGF binding protein 3 (IGFBP-3) (Figure 5B), which blocks binding of IGFs to their cognate receptors [30].

Estrogen is known to modulate IGF-I signaling in breast cancer, and ER induces IGF-IR and IGF-II expression [31]. The IGF-IR and InsR gene promoters also contain binding sites for the FoxO transcription factors, including FoxO3a, which is inhibited when phosphorylated by AKT [32]. FoxO proteins can bind directly to insulin-responsive sequences (IRSs), such as those found in the IGFBP-1 promoter, or IRS-like DNA-sequences [33]. Blockade of AKT inhibits FoxO3a phosphorylation, resulting in translocation of FoxO3a to the nucleus, where it regulates gene transcription. Further, FoxO3a has been shown to interact functionally with ER [34-38], prompting us to speculate that IGF-IR, IGF-I, and IGF-II are regulated by both ER and FoxO. Since AZD5363 induces FoxO3a nuclear translocation in ER+/PIK3CA mutant breast cancer cells [15] and ER mRNA in LTED cells (Figure 2A), we examined whether knockdown of ER and/or FoxO3a affects AZD5363-induced transcription of IGF-IR, InsR, and IGF ligands. siRNA-mediated knockdown was confirmed by RT qPCR (Figure 5C). Downregulation of FoxO3a or ER, either alone or in combination, abrogated AZD5363-mediated induction of IGF-IR, IGF-I, IGF-II and ER mRNA (Figure 5C-D). Knockdown of FoxO3a, but not ER, inhibited the induction of InsR mRNA following treatment with AZD5363 (Figure 5D). This result was expected, since InsR is not ER-regulated. These results suggest that the AZD5363-induced upregulation of IGF-IR, IGF-I, and IGF-II is dependent on ER and FoxO3a, whereas upregulation of InsR is dependent on FoxO3a.

**Figure 6:**
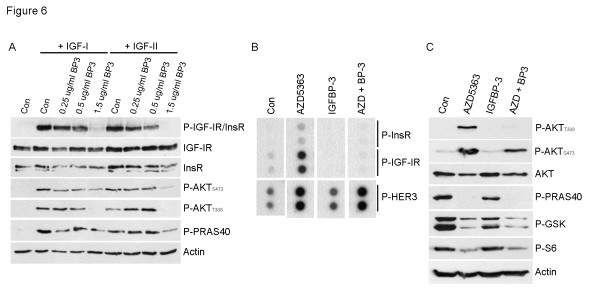
**IGFBP-3 blocks AZD5363-induced phosphorylation of IGF-IR/InsR and AKT**. **A**) MCF-7/LTED cells were treated overnight with serum-free medium ± 1.5 µg/ml IGFBP-3, followed by treatment for 15 minutes ± 100 ng/ml IGF-I or IGF-II. Protein lysates were analyzed by immunoblot using the indicated antibodies. **B**) MCF-7/LTED cells in 10% DCC-FBS were treated ± 1.5 µg/ml recombinant IGFBP-3 for 1 hour followed by 2 µM AZD5363 for 24 hours. Cell lysates (0.5 mg) were prepared and analyzed by phospho-RTK arrays. **C**) MCF-7/LTED cells in 10% DCC-FBS were treated ± 1.5 µg/ml IGFBP-3 for 1 hour followed by 2 µM AZD5363 for 24 hours. Protein lysates were prepared and analyzed by immunoblot using the indicated antibodies. DCC-FBS, dextran/charcoal-treated fetal bovine serum; IGF, insulin-like growth factor; LTED, long-term estrogen deprivation.

We then postulated that the phosphorylation of IGF-IR/InsR upon inhibition of AKT would be inhibited by blocking ligand binding to receptors with IGFBP-3. Treatment of MCF-7/LTED cells with IGFBP-3 inhibited IGF-I and IGF-II-induced phosphorylation of IGF-IR/InsR, as well as AKT (Figure 6A). IGFBP-3 also blocked AZD5363-induced phosphorylation of the IGF-IR and InsR, but not HER3 (Figure 6B). Further, IGFBP-3 completely blocked the AZD5363-induced increase in T308 P-AKT and partially that of S473 P-AKT (Figure 6C), suggesting IGF blockade inhibited PIP3 production and AKT tethering to the plasma membrane. This result suggests that the increase in IGF-IR/InsR ligands was causal to the phosphorylation of IGF-IR/InsR and AKT upon inhibition of AKT with AZD5363.

### Pharmacological inhibition of IGF-IR/InsR enhances the anti-tumor effect of AZD5363 *in vivo*

**Figure 7:**
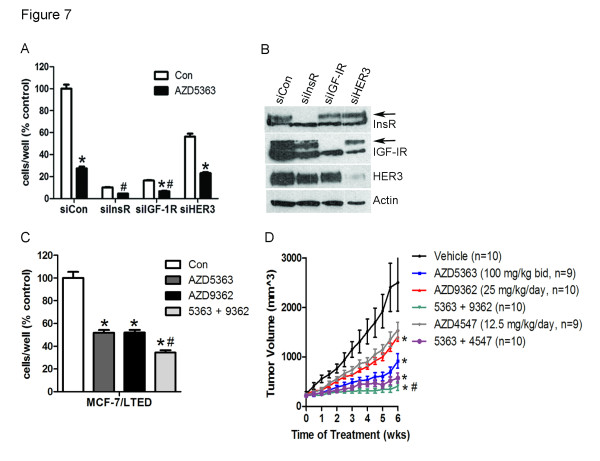
**Inhibition of IGF-IR/InsR enhances the anti-tumor effect of the AKT inhibitor AZD5363**. **A**) MCF-7 cells were transfected with siRNA specific for a non-silencing control (siCon), InsR, IGF-IR or HER3 and re-seeded the next day for assessment of growth in monolayer (A) or immunoblot analyses (**B**). In A, cells were treated with 10% DCC-FBS ± 2 µM AZD5363 and counted after five days. Data are presented as % of control; mean ± SEM (*n *= 3; *P <0.0001 versus each Con, #*P *<0.01 versus siCon + AZD; two-way ANOVA). In B, cells were grown in 10% DCC-FBS and harvested three days later; lysates were analyzed by immunoblot using the indicated antibodies. **C**) MCF-7/LTED cells in 10% DCC-FBS were treated ± 2 µM AZD5363 or 1 µM AZD9362. Media and drugs were replenished every three days. Cells were counted after five days. Data are presented as % of control; mean ± SEM (*n *= 2; *P <0.0001 versus Con, #*P *<0.05 versus 5363 or 9362; one-way ANOVA). **D**) Mice bearing MCF-7 xenografts ≥150 mm^3 ^were randomized to the indicated treatments. Data are presented as mean tumor volume ± SEM (*P <0.05 versus vehicle; ^#^*P *= 0.0041 versus AZD5363, *t*-test). ANOVA, analysis of variance; DCC-FBS, dextran/charcoal-treated fetal bovine serum; IGF, insulin-like growth factor; LTED, long-term estrogen deprivation; SEM, standard error of the mean.

Since LTED cells compensate for AKT inhibition by upregulating IGF-IR/InsR activity (Figure 3), we examined whether inhibition of this pathway sensitizes to the AKT inhibitor. siRNA-mediated knockdown of IGF-IR or InsR, but not HER3, significantly enhanced the growth inhibitory effects of AZD5363 in MCF-7 cells (Figure 7A,B). We next investigated the effects of the reversible, ATP-competitive dual IGF-IR/InsR TKI AZD9362. AZD9362 inhibits autophosphorylation of IGF-IR in fibroblasts from an IGF-IR knockout mouse stably transfected with human IGF-IR (IC_50 _of 48 nM), as well as autophosphorylation of InsR in CHO cells transfected with human InsR (IC_50 _of 186 nM; AstraZeneca, unpublished data). Treatment with AZD9362 also significantly sensitized cells to the AKT inhibitor (Figure 7C), suggesting that LTED cells compensate for AKT inhibition by upregulating IGF-IR/InsR kinase activity. Since inhibition of AKT with AZD5363 upregulated both IGF-IR/InsR and FGFR activity *in vivo *(Figure 3D-E), we next assessed the combination of AZD5363 with AZD9362 or with the FGFR TKI AZD4547 against MCF-7 xenografts. AZD4547 potently inhibits the FGFR1, 2 and 3 tyrosine kinases (IC_50 _of 0.2, 2.5 and 1.8 nM, respectively), but displays weaker activity against FGFR4 (IC_50 _of 165 nM) [19]. Treatment with AZD5363 or AZD9362 but not the FGFR antagonist inhibited tumor growth compared to vehicle (*P *<0.05; Figure 7D). This was consistent with the report that 30 µM of AZD4547 did not affect MCF-7 proliferation *in vitro *[19]. Addition of AZD4547 to AZD5363 modestly increased its anti-tumor effect, albeit not significantly. However, combined treatment with AZD5363 and the InsR/IGF-IR inhibitor AZD9362 was significantly superior to AZD5363 alone (*P *= 0.004), inducing a complete tumor regression in one mouse (Figure 7D). Overall, the drug combinations were well-tolerated with <10% weight loss [see Additional file 2, Figure S9]. These results suggest that combined inhibition of AKT and IGF-IR/InsR is more effective against MCF-7 xenografts established in ovariectomized mice.

## Discussion

PI3K/AKT/mTOR pathway activation has been implicated in endocrine resistance in breast cancer [9,10,12-14]. High AKT expression in breast tumors has also been associated with a poor response to antiestrogen therapy [39,40]. In support of this notion, we show herein that the catalytic AKT inhibitor AZD5363 inhibited the growth of ER+ human breast cancer cells with acquired resistance to estrogen deprivation and prevented the emergence of hormone-independent cells. Inhibition of AKT suppressed growth of MCF-7 xenografts in ovariectomized mice and in a patient-derived breast cancer resistant to tamoxifen and fulvestrant. Combined inhibition of ER and AKT was more effective than each intervention alone. AKT inhibition resulted in feedback upregulation and activation of RTKs *in vitro *and *in vivo*, including IGF-IR, InsR, HER3 and FGFRs. Inhibition of IGF-IR/InsR or PI3K abrogated AKT PH-GFP membrane localization and AKT phosphorylation following treatment with AZD5363. Inhibition of AKT resulted in upregulation of ER- and FoxO-dependent IGF-IR, IGF-I, and IGF-II. Treatment with IGFBP-3 blocked the AZD5363-induced phosphorylation of IGF-IR/InsR and AKT, suggesting that the induced ligands activated IGF-IR/InsR. Finally, inhibition of IGF-IR/InsR enhanced the antitumor effect of the AKT inhibitor both *in vitro *and *in vivo*.

Inhibition of AKT with AZD5363 resulted in upregulation and activation of several RTKs. Others have seen upregulation of RTKs upon inhibition of the PI3K/AKT/mTOR pathway, including HER3 [22-24]. We show that this feedback reactivation also occurs in antiestrogen-resistant breast cancer cells and xenografts using a catalytic inhibitor of AKT. AZD5363 treatment resulted in prominent upregulation of IGF-IR/InsR expression and activity both *in vitro *and *in vivo *(Figure 3). In turn, InsR/IGF-IR stimulated membrane localization and phosphorylation of AKT in T308 likely as a result of increased production of PIP3. Indeed, inhibition of IGF-IR/InsR or PI3K abrogated AKT PH-GFP membrane localization and P-AKT following treatment with AZD5363 (Figure 4). While the increase in InsR/IGF-IR levels can be explained by increased FoxO-dependent mRNA transcription (Figure 5D; [22,23]), it is less clear why receptor phosphorylation would increase following inhibition of AKT. However, we observed that upon inhibition of AKT, IGF-I and IGF-II mRNA were increased whereas IGFBP-3 mRNA levels were reduced (Figure 5A-B), thus revealing a previously unreported autocrine loop. Treatment with IGFBP-3 blocked AZD5363-induced phosphorylation of IGF-IR/InsR and AKT (Figure 6), suggesting that increased IGF-IR/InsR ligand production and activation of IGF-IR/InsR activates PI3K upstream AKT.

Inhibition of the PI3K/AKT pathway using AZD5363 or BKM120 induced ERα expression (Figure 2A; Additional file 2, Figure S3). In agreement with our data, Guo and colleagues reported that constitutively active AKT reduces ERα expression, whereas AKT inhibition increases ERα levels [35]. Knockdown of FoxO3a reduced ERα mRNA and limited the AZD5363-mediated induction of ERα (Figure 5C), suggesting that its compensatory upregulation may be dependent on FoxO3a. In support of this, Guo and colleagues reported that expression of a dominant negative FoxO3a decreased ERα levels in MCF-7 cells [35]. Further, FoxO3a has been shown to transactivate ERα[35,38]. In contrast, others have shown that FoxO3a negatively regulates ER transcriptional activity [34,36,37]. These differing reports may be due to the use of different cellular systems and the presence or absence of estrogen. Importantly, we also identified a novel role for FoxO3a in regulating AZD5363-induced ER, IGF-I and IGF-II transcription. Further, AZD5363-induced upregulation of IGF-IR, IGF-I and IGF-II mRNA was dually regulated by FoxO3a and ER (Figure 5D). We propose that inhibition of AKT induces FoxO3a nuclear translocation [15] and transcriptional activation (Figure 5C), leading to increased ER, InsR, IGF-IR, IGF-I and IGF-II expression. ER also regulates IGF-IR, IGF-I and IGF-II transcription, ultimately leading to enhanced phosphorylation of IGF-IR/InsR and AKT.

Compensation for AKT inhibition through InsR/IGF-IR signaling has therapeutic implications in cancer. Although treatment with AZD5363 upregulated HER3 mRNA and protein levels (Figures 3A-B), knockdown of HER3 did not sensitize to AZD5363 treatment in MCF-7 cells (Figure 7A). Consistent with this result, treatment with the EGFR/HER2 dual kinase inhibitor lapatinib, which blocks HER3 phosphorylation in MCF-7 cells, does not suppress P-AKT in MCF-7 cells [9]. These data suggest that HER3 does not appreciably activate PI3K in these cells. In contrast, RNAi-mediated knockdown or pharmaceutical inhibition of IGF-IR/InsR sensitized breast cancer cells to the AKT inhibitor (Figures 7A,C). We have previously identified IGF-IR/InsR signaling as a mechanism of escape from hormone dependence in ER+ breast cancer [41]. In keeping with this, inhibition of IGF-IR/InsR with AZD9362 suppressed MCF-7 xenograft growth in ovariectomized mice devoid of estrogen supplementation (Figure 7D). Importantly, treatment with AZD9362 also enhanced the anti-tumor effects of the AKT inhibitor against MCF-7 xenografts (Figure 7D), suggesting that combined inhibition of IGF-IR/InsR and AKT should be more effective than either agent alone in treating ER+ breast cancers that adapt to estrogen deprivation. We also showed that long-term treatment with the pan-PI3K inhibitor BKM120 increased IRS-1 levels in T47D cells [see Additional file 2, Figure S3], providing an additional rationale for combining PI3K/AKT and IGF-IR/InsR antagonists. Addition of the FGFR inhibitor AZD4547 also increased the anti-tumor effects of AZD5363 *in vivo*, albeit modestly (Figure 7D). FGFR1 amplification has been shown to drive endocrine therapy resistance, and patients with ER+ positive tumors that overexpress FGFR1 exhibit a shorter relapse-free survival after adjuvant tamoxifen [42]. Thus, combined inhibition of AKT with FGFR in the setting of antiestrogen resistance warrants further investigation.

## Conclusions

Upregulation of IGF-IR/InsR and their ligands compensates for AKT inhibition in breast cancer cells with acquired resistance to estrogen deprivation, implying that AKT inhibitors may have limited clinical activity in endocrine-resistant breast cancers when used as single agents. Inhibition of the IGF-IR/InsR signaling pathway enhanced the action of AZD5363 against estrogen-deprived breast cancers, suggesting that combined treatment with an AKT inhibitor and a dual IGF-IR/InsR TKI merits evaluation as a potential treatment for endocrine-resistant breast cancer.

## Abbreviations

AIs: aromatase inhibitors; DCC-FBS: dextran/charcoal-treated FBS; E2: 17β-estradiol; ER+: estrogen receptor α-positive; FBS: fetal bovine serum; FGFR: fibroblast growth factor receptor; FoxO: forkhead box class O; IGF-IR: insulin-like growth factor-I receptor; IHC: immunohistochemistry; IMEM: improved minimum essential medium; InsR: insulin receptor; IRSs: insulin-responsive sequences; LTED: long-term estrogen deprivation; PH: pleckstrin homology; PI3K: phosphatidylinositol-3 kinase; *PIK3CA*: PI3K catalytic subunit p110α; *PIK3R1: PI3K *regulatory subunit p85α;*PIK3CB*: PI3K catalytic subunit p110β; PIP_2_: phosphatidylinositol 4,5-bisphosphate; PIP_3_: phosphatidylinositol 3,4,5-trisphosphate; PR: progesterone receptor; qPCR: quantitative polymerase chain reaction; RTKs: receptor tyrosine kinases; siRNA: small interfering RNA; TKI: tyrosine kinase inhibitor.

## Competing interests

BRD is an employee of AstraZeneca. EMF, MGK, TWM and CLA declare that they have no competing interests.

## Authors' contributions

EMF performed laboratory work, nude mouse xenograft experiments and data analysis, as well as wrote the manuscript. MGK scored the immunohistochemistry (IHC) sections in Figure S4 in Additional file 2. TWM performed the immunoblot analysis in Figure S3 in Additional file 2 and provided scientific advice. CLA and BRD conceived and designed the study. CLA prepared the manuscript with EMF and allocated funding for the work. CLA and BRD critically revised the manuscript and provided scientific direction. All authors read and approved the final manuscript.

## Supplementary Material

Additional file 1 **Supplementary materials and methods for Supplementary figures**. A pdf file presenting the Supplementary materials and methods. Click here for file

Additional file 2 **Supplementary figures S1-S9**. Figure S1 showing inhibition of AKT with AZD5363 reduces phosphorylation of AKT/TORC1 substrates in ER+ breast cancer cells. Figure S2 showing inhibition of AKT with AZD5363 prevents the emergence of hormone-independent ER+ breast cancer cells. Figure S3 showing inhibition of PI3K with BKM120 upregulates ER expression and activity. Figure S4 showing treatment with AZD5363 and fulvestrant synergistically inhibits proliferation *in vivo*. Figure S5 showing AKT inhibition suppresses the growth of HBCx-3 ER+ luminal B breast cancer xenografts. Figure S6 showing the Src inhibitor dasatinib suppresses AZD5363-induced upregulation of HER3 phosphorylation and enhances its growth inhibitory effects. Figure S7 showing inhibition of AKT is followed by phosphorylation of multiple RTKs. Figure S8 showing inhibition of AKT with AZD5363 upregulates IGF-I and IGF-II protein levels. Figure S9 showing mice exhibit minimal weight loss when treated with pharmacological inhibitors. Click here for file
