# Supplementary material for: Autocrine IGF-I/insulin receptor axis compensates for inhibition of AKT in ER-positive breast cancer cells with resistance to estrogen deprivation
Source: Breast Cancer Res. 2013 Jul 11;15(4):R55. doi: 10.1186/bcr3449 (PMC3979036; doi:10.1186/bcr3449)
Supplement: Additional file 1 — Supplementary materials and methods for Supplementary figures. A pdf file presenting the Supplementary materials and methods. [file bcr3449-S1.PDF]

Fox *et al*. **Additional file 1**

Autocrine IGF-I/Insulin receptor axis compensates for inhibition of AKT in ER-positive breast cancer cells with acquired resistance to estrogen deprivation

Supplementary materials and methods for Supplementary figures

HBCx-3 breast cancer explant model. An explant (HBCx-3) from a post-menopausal woman with grade 3 invasive breast carcinoma who received no previous treatment and had no metastases was established as described previously [1]. Tumors were transplanted into female athymic nude mice (Hsd: Athymic nude – Fox1nu). When tumors reached 1-2 cm^3, the mice were sacrificed. The xenografts were excised and necrotic portions removed with the remainder cut into fragments of ~20 mm^3. Recipient mice were anaesthetized with kentamine/zylazine, the skin aseptized with chlorhexidine, and an incision was made into the interscapular region. A single tumor fragment was placed into the s.c. space and the skin closed with clips. When the tumors reached ≥60 mm^3, mice were randomized to treatment with vehicle, AZD5363 (150 mg/kg bid p.o. or 100 mg/kg bid p.o.), tamoxifen (4 mg/kg x3 each week p.o.), or fulvestrant (150 mg/kg x 3 each week i.p.). Tumor diameters were measured using calipers twice weekly. Tumors were harvested and flash-frozen in liquid nitrogen, or fixed for paraffin-embedding and IHC.

Mouse xenograft experiments. Animal experiments were approved by the Vanderbilt Institutional Animal Care and Use Committee. Female ovariectomized athymic 4- to 5-week old Balb/c mice (Harlan Sprague Dawley) were implanted s.c. with a 14-day-release 17β-estradiol pellet (0.17 mg; Innovative Research of America) in the dorsal space. The next day, 10^7 MCF-7 cells suspended in IMEM and mixed with matrigel (BD Biosciences) at 1:1 ratio were injected s.c. into the right flank of each mouse. After >2 weeks, mice bearing tumors ≥150 mm^3 were

randomized to treatment with vehicle [25% (2-Hydroxypropyl)-β-cyclodextrin], AZD5363 (150 or 100 mg/kg bid p.o.), fulvestrant (5 mg/wk i.p.), AZD9362 (25 mg/kg/day p.o.) or AZD4547 (12.5 mg/kg/day p.o.). Combining 150 mg/kg/day AZD5363 with AZD9362 and AZD4547 resulted in excessive toxicity, so a lower dose of AZD5363 (100 mg/kg/day p.o.) was used in this experiment. Tumor diameters were measured using calipers twice weekly (volume = width^2 x length/2). Mice were weighed daily and those that lost >10% of body weight were given a 1-ml i.p. injection of Dextrose-Free Lactated Ringer's Injection USP (Baxter Healthcare Corp.). Tumors were harvested 1 h or 4 h after the last dose of AZD5363 or 24 h after the last dose of fulvestrant and flash-frozen in liquid nitrogen or fixed in 10% formalin for paraffin-embedding. Frozen tumors were homogenized using the TissueLyser II (Qiagen). Tumor lysates were prepared and analyzed by immunoblot. Five-μm paraffin sections were used for IHC using a Ki67 antibody (Biocompare) as described in [2]. Tumor sections were studied on a light microscope with an ocular magnification of 400×. Average intensity of tumor cell staining was calculated as a membrane histoscore as described previously [3]. A trained pathologist (M.G.K.) blinded to the type of treatment scored the sections.

IGF-I and IGF-II ELISAs. 3x10^5 (MCF-7) or 5x10^5 (ZR75-1, MDA-361, HCC-1428) LTED cells were plated in each well of a 6-well plate and treated with 1.5 ml of 10% DCC-FBS ± 2 μM AZD5363 for 24 or 48 h. Cell culture supernatants were collected and centrifuged at 2000 rpm for 20 min at 4 º Celsius. ELISAs for IGF-I (Human IGF-I Quantikine ELISA Kit, R&D Systems) and IGF-II (Human IGF2 ELISA Kit, Abnova) were performed using the cell supernatants according to the manufacturer's protocol. Concentrations in pg/ml were calculated using a standard curve. Recombinant IGF-I (4 or 6 ng/ml) or IGF-II (4 or 6 ng/ml) (R&D Systems) were used as controls. The standard curve for the IGF-I kit ranged from 0-6 ng/ml, and the standard curve for the IGF-II kit ranged from 0-4 ng/ml. The experimental concentrations were within the range of the standard curve.

References

1. Marangoni E, Vincent-Salomon A, Auger N, Degeorges A, Assayag F, de Cremoux P, de Plater L, Guyader C, De Pinieux G, Judde JG, Rebucci M, Tran-Perennou C, Sastre-Garau X, Sigal-Zafrani B, Delattre O, Dieras V, Poupon MF: **A new model of patient tumor-derived breast cancer xenografts for preclinical assays**. *Clin Cancer Res* 2007, **13**:3989-3998.

2. Fox EM, Miller TW, Balko JM, Kuba MG, Sanchez V, Smith RA, Liu S, Gonzalez-Angulo AM, Mills GB, Ye F, Shyr Y, Manning HC, Buck E, Arteaga CL: **A kinome-wide screen identifies the insulin/IGF-I receptor pathway as a mechanism of escape from hormone dependence in breast cancer**. *Cancer Res* 2011, **71**:6773-6784.

3. Goulding H, Pinder S, Cannon P, Pearson D, Nicholson R, Snead D, Bell J, Elston CW, Robertson JF, Blamey RW, et al.: **A new immunohistochemical antibody for the assessment of estrogen receptor status on routine formalin-fixed tissue samples**. *Hum Pathol* 1995, **26**:291-294.
