# Supplementary material for: Autocrine IGF-I/insulin receptor axis compensates for inhibition of AKT in ER-positive breast cancer cells with resistance to estrogen deprivation
Source: Breast Cancer Res. 2013 Jul 11;15(4):R55. doi: 10.1186/bcr3449 (PMC3979036; doi:10.1186/bcr3449)
Supplement: Additional file 2 — Supplementary figures S1-S9. Figure S1 showing inhibition of AKT with AZD5363 reduces phosphorylation of AKT/TORC1 substrates in ER+ breast cancer cells. Figure S2 showing inhibition of AKT with AZD5363 prevents the emergence of hormone-independent ER+ breast cancer cells. Figure S3 showing inhibition of PI3K with BKM120 upregulates ER expression and activity. Figure S4 showing treatment with AZD5363 and fulvestrant synergistically inhibits proliferation in vivo. Figure S5 showing AKT inhibition suppresses the growth of HBCx-3 ER+ luminal B breast cancer xenografts. Figure S6 showing the Src inhibitor dasatinib suppresses AZD5363-induced upregulation of HER3 phosphorylation and enhances its growth inhibitory effects. Figure S7 showing inhibition of AKT is followed by phosphorylation of multiple RTKs. Figure S8 showing inhibition of AKT with AZD5363 upregulates IGF-I and IGF-II protein levels. Figure S9 showing mice exhibit minimal weight loss when treated with pharmacological inhibitors. [file bcr3449-S2.PDF]

Fox *et al*. **Additional file 2**

Autocrine IGF-I/Insulin receptor axis compensates for inhibition of AKT in ER-positive breast cancer cells with acquired resistance to estrogen deprivation

Supplementary figures S1-S9

Figure S1

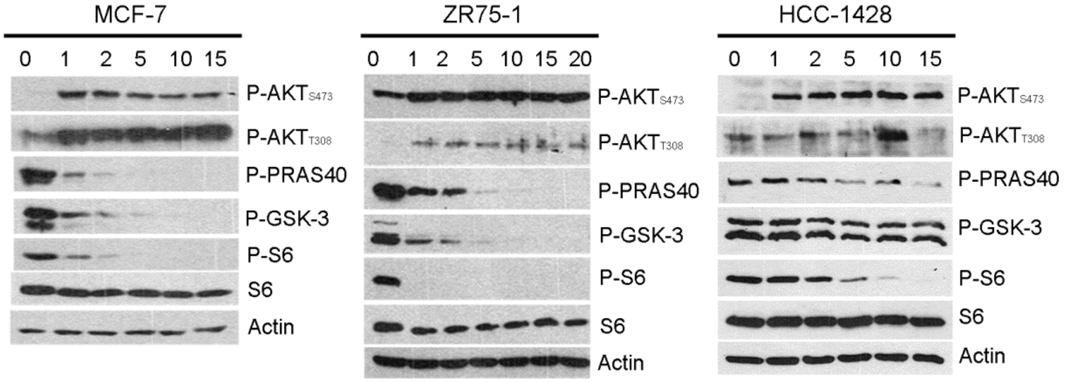

Figure S1. Catalytic AKT inhibitor AZD5363 reduces phosphorylation of AKT/TORC1 substrates in ER+ breast cancer cells. Cells were treated with 10% FBS ± 0-20 µM AZD5363 for 24 h. Protein lysates were analyzed by immunoblot using the indicated antibodies.

Figure S2

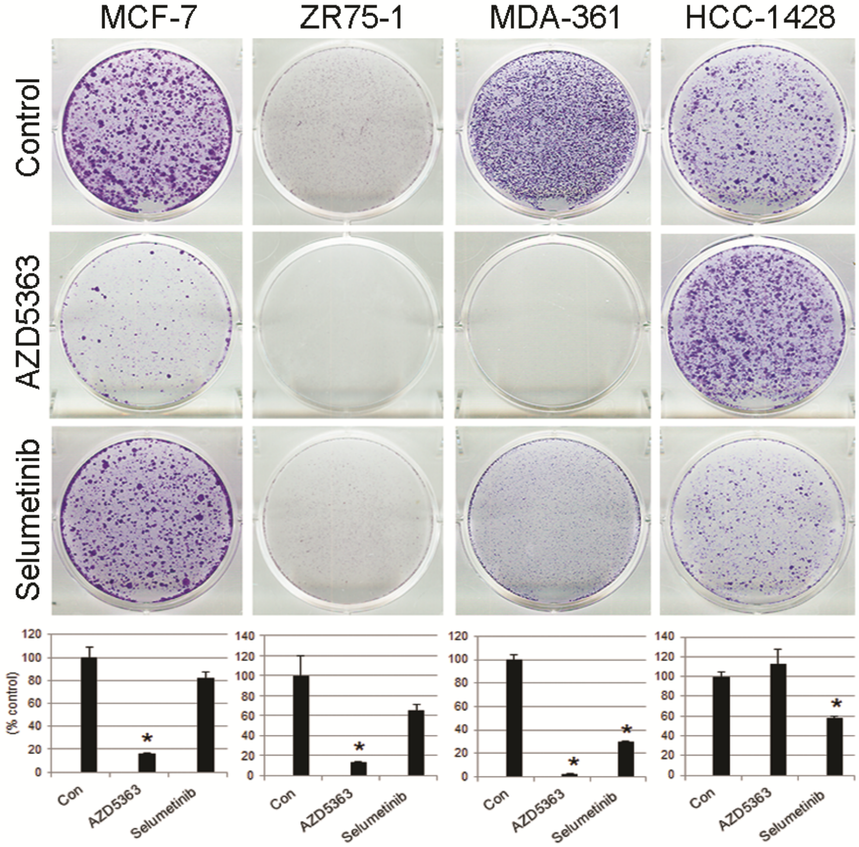

Figure S2. Inhibition of AKT with AZD5363 prevents the emergence of hormone-independent ER+ breast cancer cells. Parental cells in 10% DCC-FBS were treated ± 0.4 µM AZD5363 or 1 µM selumetinib. Media and inhibitors were replenished every 3 days. When control wells reached 60-80% cell confluence [after 15 (MCF-7), 30 (ZR75-1), 25 (MDA-361) or 39 (HCC-1428) days, respectively], cells were fixed and stained with crystal violet. Representative images and quantification of integrated intensity (% control) are shown (**p*<0.05 *vs.* control, *t*-test).

Figure S3

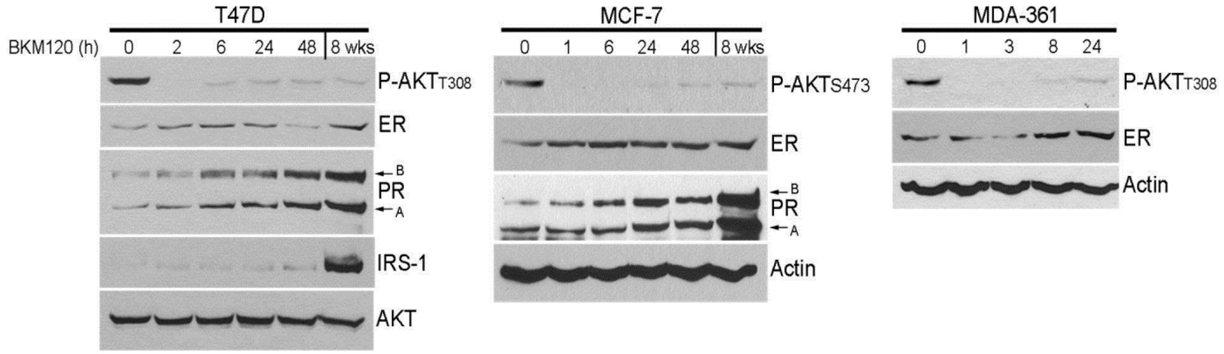

Figure S3. Inhibition of PI3K with BKM120 upregulates ER expression and activity. ER+ MCF-7, T47D, and MDA-361 breast cancer cells were treated with medium containing 10% FBS +/- 1 µM BKM120 for the indicated time points. MCF-7 and T47D cells were also selected for growth in the presence of 1 µM BKM120 for approximately 8 weeks. Protein lysates were prepared, separated by SDS-PAGE, and analyzed by immunoblot with the indicated antibodies.

Figure S4

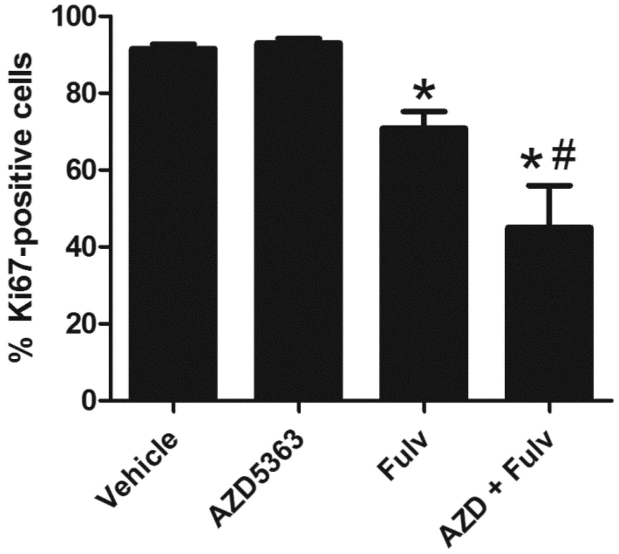

Figure S4. Treatment with AZD5363 and fulvestrant synergistically inhibits proliferation *in vivo*. MCF-7 cells were injected s.c. into athymic mice supplemented with 14-day release 17β-estradiol pellets. Mice bearing tumors ≥150 mm^3 were randomized to vehicle, AZD5363 (150 mg/kg/day bid p.o.), fulvestrant (5 mg/wk i.p.), or both drugs for six weeks. Quantification of Ki67+ tumor cell nuclei by IHC is shown (*$p<0.001$ *vs.* vehicle; # $p<0.05$ *vs*. AZD or fulv, *t*-test).

Figure S5

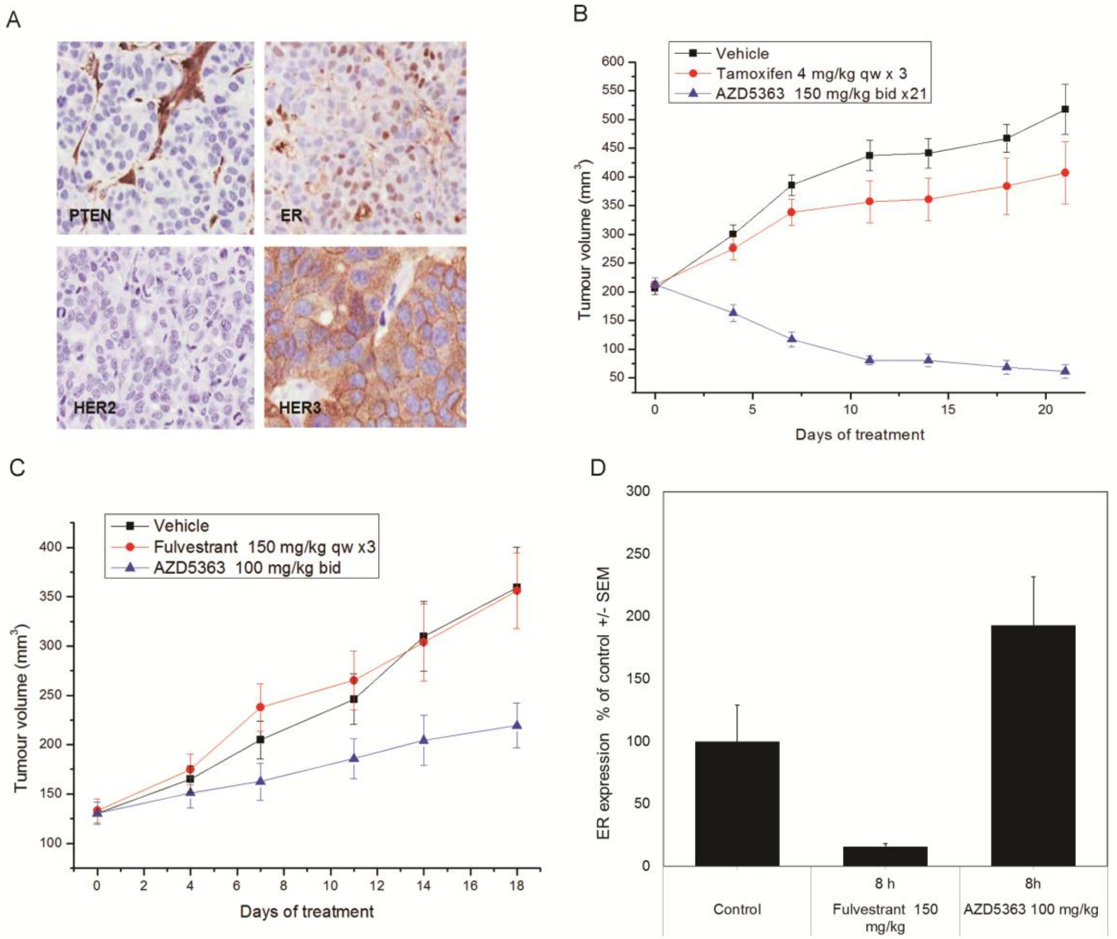

Figure S5. AKT inhibition suppresses the growth of HBCx-3 ER+ luminal B breast cancer xenografts. A) Patient derived xenografts (HBCx-3) were transplanted into nude mice as described in Methods. Representative images from IHC for PTEN, ER, HER2, and HER3 are shown. B) Mice bearing tumors 60-200 mm^3 were randomized to treatment with vehicle, AZD5363 (150 mg/kg bid p.o.), or tamoxifen (4 mg/kg x3 each week p.o.), for 21 days. Data are presented as mean tumor volume ± SEM. C) Mice bearing HBCx-3 xenografts 60-200 mm^3 were randomized to treatment with vehicle, AZD5363 (100 mg/kg bid p.o.), or fulvestrant (150 mg/kg x3 each week i.p.), for 18 days. Data are presented as mean tumor volume ± SEM. D) Xenografts from C) were homogenized and tumor lysates were analyzed by immunoblot. Quantitation of immunoblots are presented as ER expression percent of control ± SEM (n=5).

Figure S6

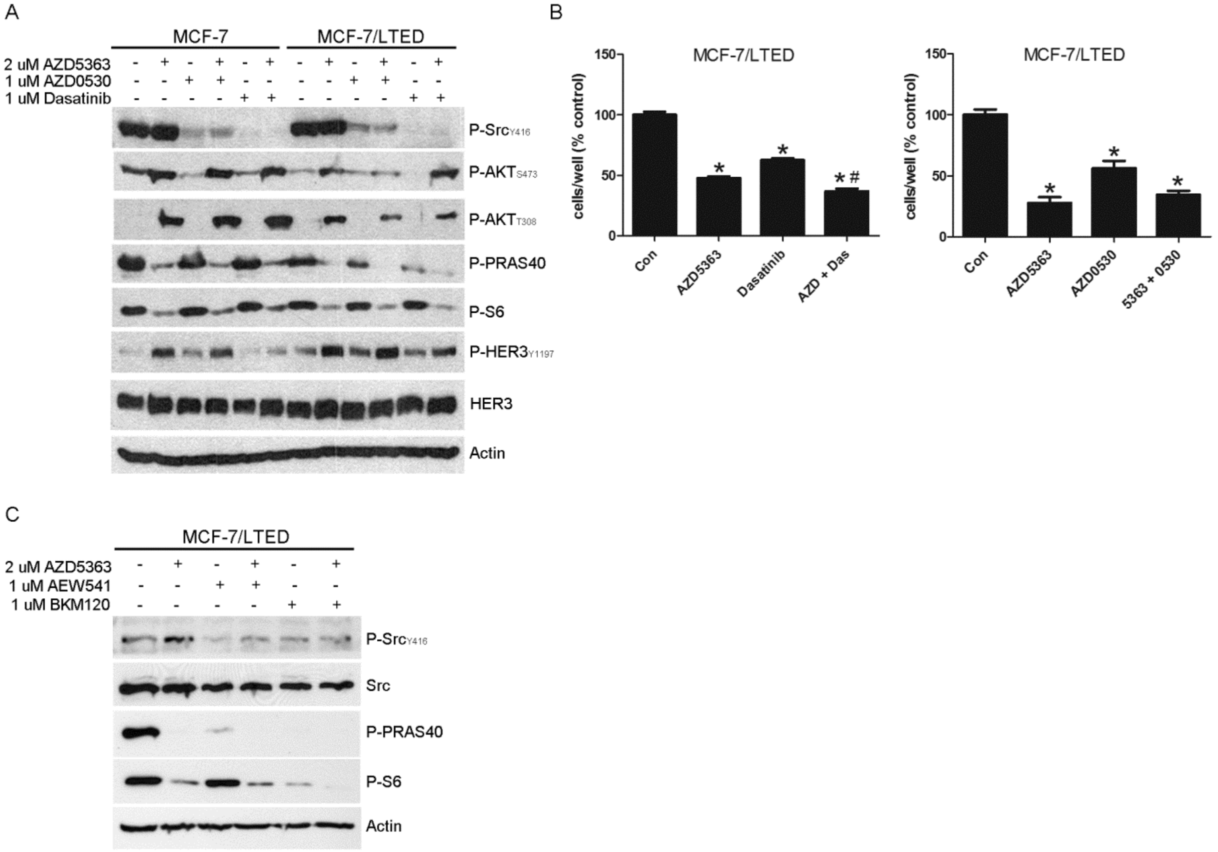

Figure S6. Src inhibitor dasatinib suppresses AZD5363-induced upregulation of HER3 phosphorylation and enhances its growth inhibitory effects. A) 2.5x10^5 MCF-7 or MCF-7/LTED cells were plated in 6-well plates. Cells in 10% DCC-FBS were pre-treated for 1 h with 1 µM AZD0530 or 1 µM dasatinib, followed by 24 h ± 2 µM AZD5363. Protein lysates were analyzed by immunoblot using the indicated antibodies. B) LTED cells were treated with 10% DCC-FBS ± 2 µM AZD5363, 1 µM AZD0530 or 1 µM dasatinib. Media and drugs were replenished every 3 days. Cells were counted after 5 days. Data are presented as percent of control; each bar, mean ± SEM (n=3; **p*<0.0001 *vs.* Con, # *p*<0.05 *vs*. AZD or Das, one-way ANOVA). C) MCF-7/LTED cells were treated with 10% DCC-FBS ± 1 µM AEW541 or BKM120 for 1 h, followed by addition of 2 µM AZD5363 for 24 h. Protein lysates were analyzed by immunoblot with the indicated antibodies.

Figure S7

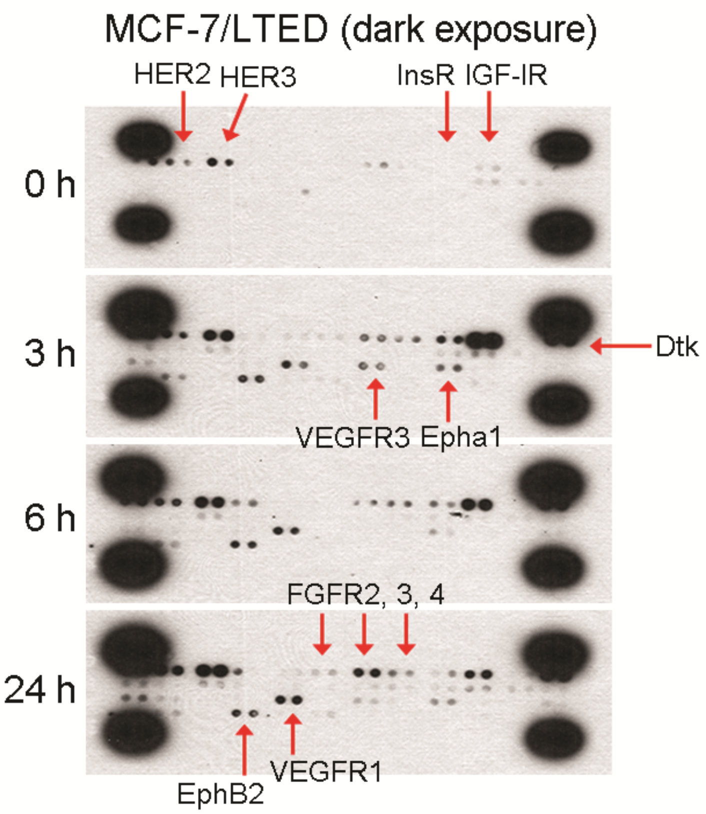

Figure S7. Inhibition of AKT is followed by phosphorylation of multiple RTKs**.** LTED cells in 10% DCC-FBS were treated ± 2 µM AZD5363 for 0, 3, 6, and 24 hr. Cell lysates were prepared and analyzed by phospho-RTK arrays as described in Methods. A darker exposure is shown here.

Figure S8

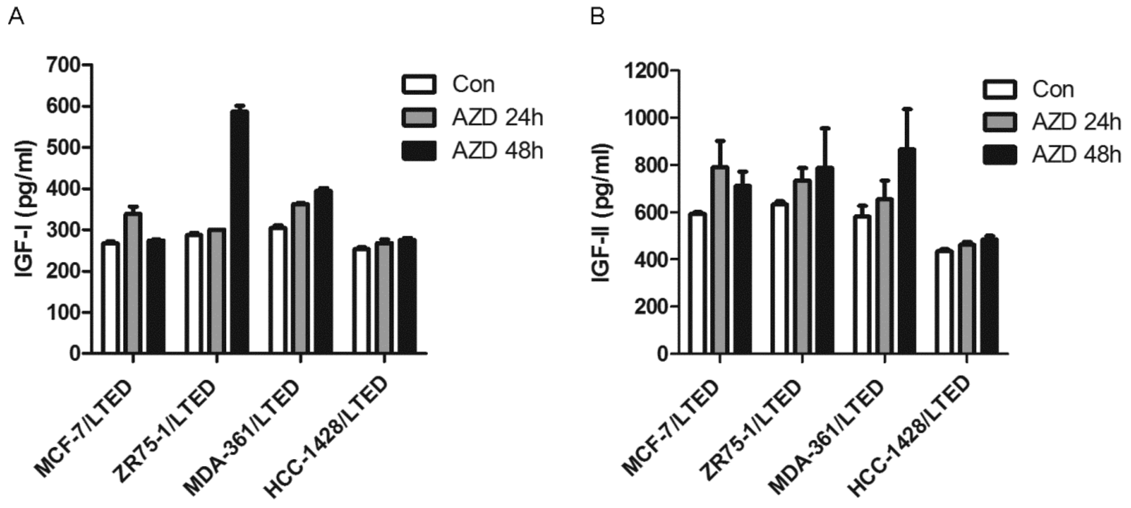

Figure S8. Inhibition of AKT with AZD5363 upregulates IGF-I and IGF-II protein levels. $3x10^5$ (MCF-7) or $5x10^5$ (ZR75-1, MDA-361, HCC-1428) LTED cells were plated in each well of a 6-well plate and treated with 1.5 ml of 10% DCC-FBS ± 2 µM AZD5363 for 24 or 48 h. Cell culture supernatants were collected and ELISAs for IGF-I (A) or IGF-II (B) were performed as described in the Methods. Concentrations in pg/ml were calculated using a standard curve. Recombinant IGF-I (4 or 6 ng/ml) or IGF-II (4 or 6 ng/ml) were used as controls (data not shown). The standard curve for the IGF-I kit ranged from 0-6 ng/ml, and the standard curve for the IGF-II kit ranged from 0-4 ng/ml. The experimental concentrations were within the range of the standard curve.

Figure S9

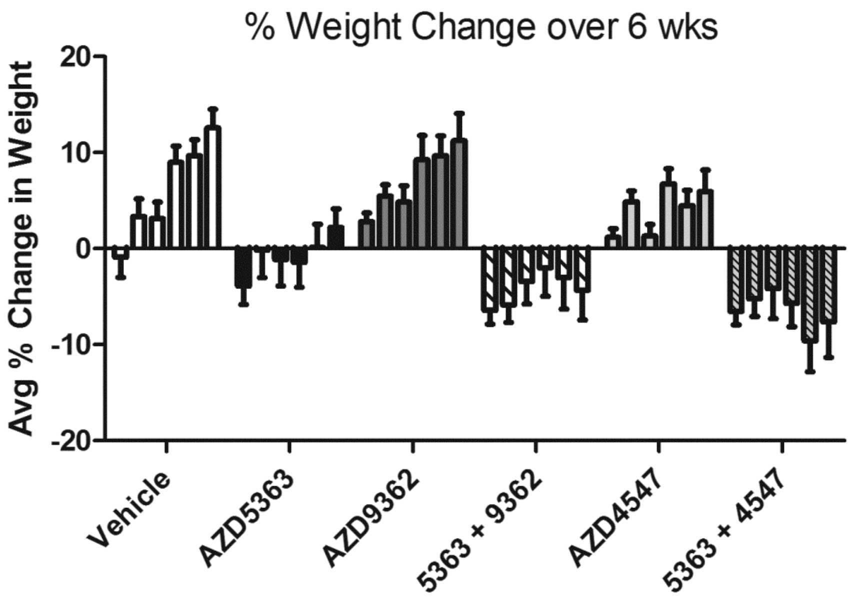

Figure S9. Mice exhibit minimal weight loss when treated with pharmacological inhibitors. MCF-7 cells were injected s.c. into athymic mice supplemented with 14-day release 17β-estradiol pellets. Mice bearing tumors ≥150 mm^3 were randomized to vehicle, AZD5363 (100 mg/kg/day bid p.o.), AZD9362 (25 mg/kg/day p.o.), AZD5363 + AZD9362, AZD4547 (12.5 mg/kg/day p.o.), or AZD5363 + AZD4547 for six weeks. Data are presented as the average percent change in weight on week 1-6 for each treatment group.
